# Projected Future Vegetation Changes for the Northwest United States and Southwest Canada at a Fine Spatial Resolution Using a Dynamic Global Vegetation Model

**DOI:** 10.1371/journal.pone.0138759

**Published:** 2015-10-21

**Authors:** Sarah L. Shafer, Patrick J. Bartlein, Elizabeth M. Gray, Richard T. Pelltier

**Affiliations:** 1 U. S. Geological Survey, Corvallis, Oregon, United States of America; 2 Department of Geography, University of Oregon, Eugene, Oregon, United States of America; 3 The Nature Conservancy, Maryland/DC, Bethesda, Maryland, United States of America; 4 U. S. Geological Survey, Denver, Colorado, United States of America; Montana State University, UNITED STATES

## Abstract

Future climate change may significantly alter the distributions of many plant taxa. The effects of climate change may be particularly large in mountainous regions where climate can vary significantly with elevation. Understanding potential future vegetation changes in these regions requires methods that can resolve vegetation responses to climate change at fine spatial resolutions. We used LPJ, a dynamic global vegetation model, to assess potential future vegetation changes for a large topographically complex area of the northwest United States and southwest Canada (38.0–58.0°N latitude by 136.6–103.0°W longitude). LPJ is a process-based vegetation model that mechanistically simulates the effect of changing climate and atmospheric CO_2_ concentrations on vegetation. It was developed and has been mostly applied at spatial resolutions of 10-minutes or coarser. In this study, we used LPJ at a 30-second (~1-km) spatial resolution to simulate potential vegetation changes for 2070–2099. LPJ was run using downscaled future climate simulations from five coupled atmosphere-ocean general circulation models (CCSM3, CGCM3.1(T47), GISS-ER, MIROC3.2(medres), UKMO-HadCM3) produced using the A2 greenhouse gases emissions scenario. Under projected future climate and atmospheric CO_2_ concentrations, the simulated vegetation changes result in the contraction of alpine, shrub-steppe, and xeric shrub vegetation across the study area and the expansion of woodland and forest vegetation. Large areas of maritime cool forest and cold forest are simulated to persist under projected future conditions. The fine spatial-scale vegetation simulations resolve patterns of vegetation change that are not visible at coarser resolutions and these fine-scale patterns are particularly important for understanding potential future vegetation changes in topographically complex areas.

## Introduction

Future climate changes may significantly affect the distribution of many plant species. There is substantial evidence from historical and paleoenvironmental records that vegetation has responded to past climate changes. In many cases, variations in climate have produced substantial changes in species distributions, including extirpations and extinctions [1,2]. As a result of the large effects of past climate change on vegetation, a great deal of effort has focused on understanding the potential effects of future climate change on vegetation ranging from individual plant species populations to global biomes [3,4]. These studies have included research on the implications of vegetation change for species and habitat conservation [5], ecosystem services [6], and potential feedbacks of vegetation change to the climate system [7].

A common method for projecting vegetation responses to climate change is the use of numerical vegetation models. These models range in complexity from climate-envelope models based on correlations between climate and species distributions to mechanistic models that simulate the physical processes involved in vegetation responses to climate change [8,9]. The different types of vegetation models have different strengths. Climate-envelope models can be used to simulate many species distributions relatively quickly and are often applied at fine spatial resolutions (e.g., 30-second and finer grids; [10]). However these correlative models do not simulate important processes governing vegetation responses to climate change, such as changes in plant water use efficiency in response to increased atmospheric CO_2_ concentrations [11] or the dynamics of important disturbance regimes, such as fire and insect and disease outbreaks. Climate-envelope models are particularly limited in their ability to simulate plant responses over time periods when the correlations between plants and the environmental variables being used to build these models significantly change. Mechanistic models, such as dynamic global vegetation models (DGVMs), do explicitly simulate a number of the physical processes that control vegetation responses to climate change, such as changes in plant water use efficiency or mortality related to changing fire regimes [8,9], although often these processes are simulated in a simplified form. Mechanistic models are frequently applied over large areas at relatively coarse spatial resolutions (e.g., 10-minute or 0.5-degree grids). As a result, the model simulations often do not resolve important finer-scale vegetation patterns that are particularly important in regions of topographic complexity where climate and soil properties may vary significantly over short distances.

**Fig 1:**
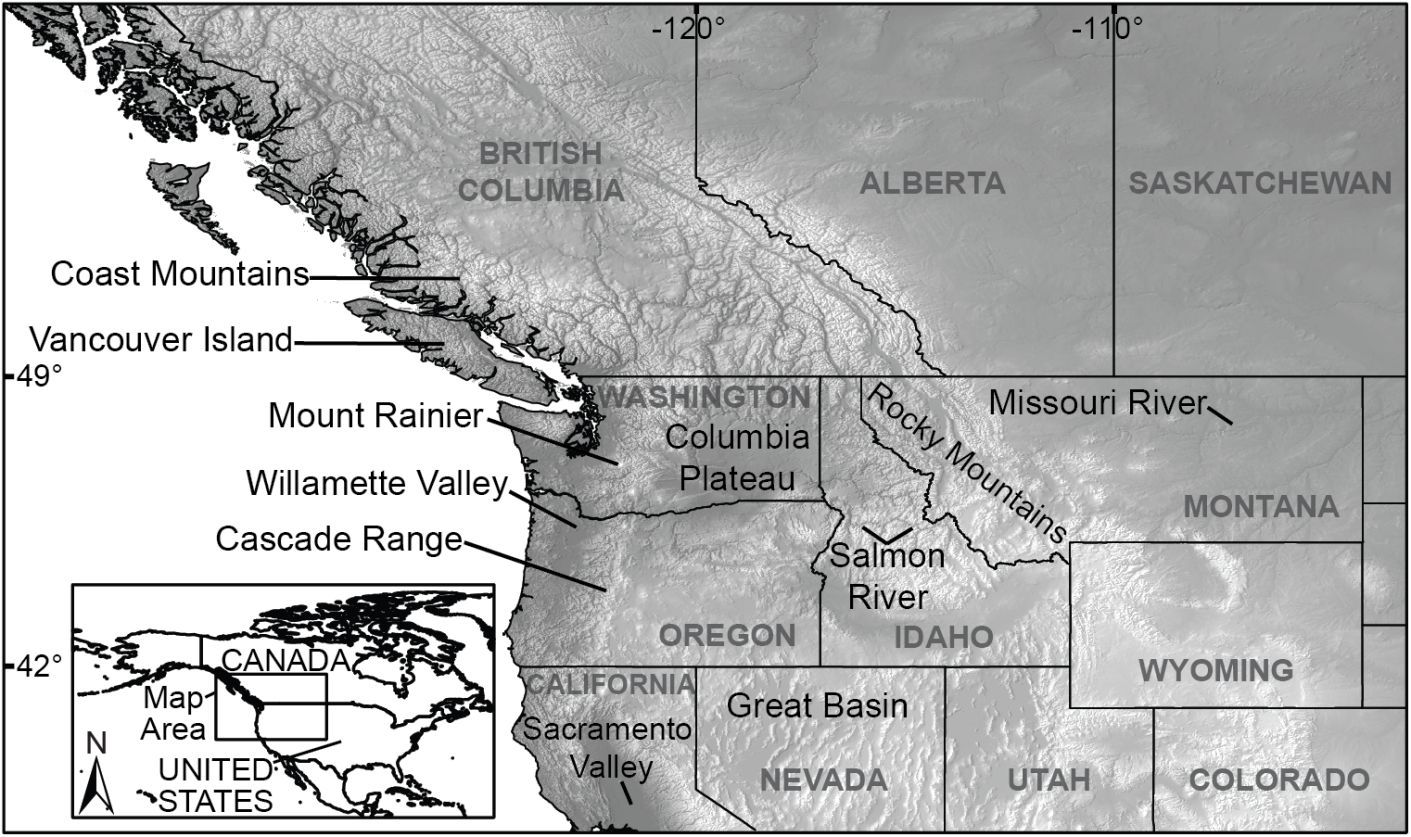
The study area and some of the physical features described in the text [15].

In this study, we used the DGVM LPJ [12] to simulate future changes in potential vegetation distributions for a large, topographically complex region of the northwest United States and southwest Canada (Fig 1). LPJ is a process-based vegetation model that has been applied at various regional scales, including East Africa [6], China [13], and western North America [14], but typically at spatial resolutions of 10-minutes or coarser. For topographically complex regions, these coarse spatial resolutions do not resolve many important topographic features and the related patterns in climate and vegetation. An example from our study area is the Cascade Range (Fig 1), a relatively narrow mountain range in western Washington and Oregon that is poorly resolved at coarse spatial resolutions (e.g., [14]), and similar examples can be found for other topographically complex regions of the globe. To be able to better resolve the influence of topography on vegetation, such as changes in vegetation along elevation gradients, we ran LPJ using a 30-second (~1-km) grid containing >6.8 million terrestrial points. This grid produces vegetation simulations with a spatial resolution that is two orders of magnitude finer than that of LPJ simulations using a 10-minute grid. We downscaled climate projections for the 21^st^ century from five coupled atmosphere-ocean general circulation models (AOGCMs) to this 30-second grid.

LPJ and LPJ-derived DGVMs have been applied to a variety of research questions, including simulations of the terrestrial carbon cycle [16], net primary productivity [13], biomass [17], ecohydrology [18], ecosystem services [6], and for both future and paleo time periods [19]. A number of studies using LPJ have focused on research questions pertaining to the carbon budget, describing potential future vegetation changes in terms of biomass or net primary productivity (e.g., [13]). However, many important ecological and biogeographical questions, such as those concerning species and habitat conservation, require information about how climate change may affect vegetation at the level of individual plant functional types (PFTs; e.g., trees, grass, shrubs) and species. Understanding potential future vegetation changes in these terms is important for addressing many conservation and natural resource management questions, such as the potential effects of climate change on threatened and endangered species or the persistence of key habitat types. Additionally, when simulating future changes in the distribution of vegetation, it is particularly important to use mechanistic models, such as LPJ, that explicitly represent vegetation responses to changes in atmospheric CO_2_ concentrations [11]. Changes in CO_2_ concentrations may affect leaf area, water use efficiency, and vegetation structure, which in turn may alter competitive interactions among species and PFTs, such as trees, grass, and shrubs that form the mosaic of forest, woodland, savanna, grassland, steppe, and shrubland in many topographically complex arid regions, including parts of our study area [20,21].

The aim of this study was to simulate vegetation responses to climate change at a fine spatial resolution across our topographically complex study area that includes vegetation ranging from xeric vegetation in the interior western United States to mesic vegetation along the Pacific Coast. LPJ was able to accurately simulate historical vegetation for many parts of the study area when compared with both potential natural vegetation data for the United States and remotely sensed land cover data, providing confidence in the model's ability to simulate vegetation at fine spatial resolutions in topographically complex regions. The simulated future vegetation changes displayed fine spatial-scale patterns of contraction, expansion, and persistence of vegetation, all mediated by the region’s topography. The results of this study improve our understanding of the potential magnitude and complexity of future vegetation changes that may occur in mountainous regions, with the caveat that many uncertainties associated with both projections of future climates and potential vegetation responses to climate change have yet to be resolved.

## Materials and Methods

### Study area grid and elevation data

The study area consists of a 30-second (0.008333-degree) grid covering a region of the northwest United States and southwest Canada from 38.0 to 58.0°N latitude and from 136.6 to 103.0°W longitude (~552,783,000 ha; Fig 1). We used 30-second elevation data from the Shuttle Radar Topography Mission version 2 (SRTM V2) data set (http://www2.jpl.nasa.gov/srtm/; [22]). For each point in our study area grid we assigned the elevation from the SRTM V2 grid cell in which our study area grid point was located.

### Soil data

For each 30-second grid point, we assigned soil data (sand [%], clay [%], soil layer thickness [mm]) from the Global Soil Data Task [23] 5-minute grid cell in which the 30-second grid point was located. This soil data set contained eight soil layers (50 mm, 50 mm, 100 mm, 100 mm, 100 mm, 200 mm, 200 mm, and 200 mm thick) representing the top meter of the soil profile. Additional soil variables (wilting point [mm], available water holding capacity [mm], and saturation [mm]) were derived for each soil layer using equations from Saxton *et al*. [24]. Although the Global Soil Data Task 5-minute soil data are spatially coarser than some soil data that are available for the Unites States (e.g., CONUS-Soil [25]), the 5-minute data have the advantage of providing data coverage for both the United States and Canada allowing simulated vegetation for these two areas to be directly comparable. Estimated fraction of plant roots in each soil layer was calculated using data from Jackson *et al*. [26].

### Historical climate data

LPJ requires time series of monthly climate data to simulate vegetation. To create the time-series data, we used three gridded climate data sets produced by the Climatic Research Unit (CRU), University of East Anglia (http://www.cru.uea.ac.uk/cru/data/hrg/), CRU CL 1.0 [27], CRU CL 2.0 [28], and CRU TS 2.1 [29], to develop regridded historical and future climate data for the study area. Local topographic (as opposed to adiabatic) lapse rates were calculated for monthly mean temperature (°C), total precipitation (mm), and mean possible sunshine (%) from the CRU CL 2.0 data set for 1961–1990 (30-year mean) using a moving-window local regression method. The values for each climate variable were interpolated to the 30-second study area grid using geographic-distance-weighted bilinear interpolation with the local lapse rates used to elevationally adjust the values of each climate variable to the elevation of each 30-second grid point except for grid points coincident with CRU CL 2.0 grid points, which were assigned the CRU CL 2.0 grid point values.

Monthly anomalies for 1901–2000 were calculated for CRU TS 2.1 temperature, precipitation and cloud data using a 1961–1990 (30-year mean) base period (also calculated from the CRU TS 2.1 data). Sunshine values were estimated from cloud cover data using methods described in Doorenbos and Pruitt [30] and Hulme *et al*. [31]. Temperature anomalies were calculated as differences (each monthly value minus the CRU TS 2.1 1961–1990 30-year mean value) and precipitation and sunshine anomalies were calculated as ratios (each monthly value divided by the CRU TS 2.1 1961–1990 30-year mean value). These anomalies (on the CRU TS 2.1 30-minute grid) were interpolated to the 30-second study area grid using geographic-distance-weighted bilinear interpolation. The 30-second interpolated anomalies for each variable were applied to the corresponding regridded CRU CL 2.0 1961–1990 (30-year mean) variable for each 30-second study area grid point to create monthly time series. In the application of anomalies, monthly sunshine data were constrained to fall within the range of 0–100%.

### Future climate data

We created downscaled projections of future climate (2001–2099) using simulations from the World Climate Research Programme’s (WCRP’s) Coupled Model Intercomparison Project phase 3 (CMIP3) multi-model dataset (http://www-pcmdi.llnl.gov/ipcc/about_ipcc.php). We used model output from the CMIP3 (Intergovernmental Panel on Climate Change (IPCC) Fourth Assessment Report) family of climate simulations to allow comparisons with existing work on projected vegetation change. We chose simulations from five AOGCMs: CCSM3 [32], CGCM3.1(T47) [33], GISS-ER [34], MIROC3.2(medres) [35], and UKMO-HadCM3 [36]. These model simulations were selected because they represented the range of CMIP3 projected temperature changes and included future projections of both increased and decreased precipitation for our study area [37,38]. Each of these models had simulations produced under the IPCC Special Report on Emissions Scenarios (SRES) A2 greenhouse gases emissions scenario [39], which produces temperature increases for 2070–2099 that overlap those produced by the more recent CMIP phase 5 (CMIP5) RCP 8.5 scenario [40]. Simulations from the CMIP5 archive (IPCC Fifth Assessment Report (AR5)) differ little from those in the earlier collection (IPCC AR5 Working Group 1 Technical Summary, Box TS.6 [38]). All five AOGCMs simulated seasonal future temperature increases across the study area for 2070–2099 (Table 1). There was less agreement among the AOGCM simulations as to seasonal precipitation changes for 2070–2099. For December-February, March-May, and September-November, all five AOGCMs simulated future precipitation increases for the study area. For June-August, GISS-ER, MIROC3.2(medres), and UKMO-HadCM3 simulated mean precipitation decreases and CCSM3 and CGCM3.1(T47) simulated small mean precipitation increases for the study area (Table 1). As the standard deviations in Table 1 indicate, all the simulations projected decreases in June-August precipitation for parts of the study area.

**Table 1:** Mean (±SD) seasonal changes for 2070–2099 (30-year mean) relative to 1961–1990 (30-year mean) for the study area as simulated by five coupled atmosphere-ocean general circulation models (AOGCMs) under the A2 greenhouse gases emissions scenario.

	Mean Temperature (°C)				Total Precipitation (%)			
AOGCM	Dec-Feb	Mar-May	Jun-Aug	Sep-Nov	Dec-Feb	Mar-May	Jun-Aug	Sep-Nov
CCSM3	5.1 (1.2)	4.2 (0.5)	5.5 (1.0)	4.5 (0.6)	9.4 (11.3)	13.8 (16.7)	0.6 (32.6)	6.0 (13.9)
CGCM3.1(T47)	4.8 (0.7)	3.6 (0.4)	4.3 (0.6)	3.5 (0.3)	27.5 (7.4)	24.7 (18.7)	3.8 (11.6)	18.6 (10.1)
GISS-ER	1.7 (0.4)	1.6 (0.7)	4.0 (1.0)	2.5 (0.6)	5.1 (6.0)	7.1 (14.8)	-17.4 (16.1)	20.0 (14.1)
MIROC3.2(medres)	5.3 (1.0)	5.0 (1.3)	5.9 (0.9)	5.2 (0.7)	20.2 (17.3)	8.5 (14.3)	-17.0 (14.3)	8.3 (14.4)
UKMO-HadCM3	3.1 (0.8)	3.3 (0.6)	6.1 (1.1)	4.8 (0.6)	26.0 (16.7)	6.7 (15.5)	-2.5 (26.2)	11.4 (17.0)

AOGCMs: CCSM3 [32], CGCM3.1(T47) [33], GISS-ER [34], MIROC3.2(medres) [35], UKMO-HadCM3 [36]

We downscaled the AOGCM future climate simulations to the 30-second study area grid by calculating anomalies of monthly temperature (°C), precipitation (mm), and cloud cover (percent) on the AOGCM grid using each future simulation’s corresponding 20^th^ century climate simulation (identified in the CMIP3 documentation at http://www-pcmdi.llnl.gov/ipcc/about_ipcc.php) to create a 1961–1990 30-year mean base period. Temperature anomalies were calculated as differences (AOGCM future monthly temperature values for each month minus the AOGCM 20^th^ century 1961–1990 30-year mean temperature for the same month). Precipitation and cloud cover anomalies were calculated as ratios (AOGCM future monthly precipitation or cloud cover values divided by the AOGCM 20^th^ century 1961–1990 30-year mean precipitation or cloud cover values for the corresponding month). The anomalies were interpolated to the 30-second study area grid using geographic-distance-weighted bilinear interpolation. The interpolated anomalies were applied to the regridded CRU CL 2.0 1961–1990 30-year mean temperature and precipitation data and CRU CL 1.0 1961–1990 30-year mean cloud cover data to create time series of future monthly climate values [41]. The cloud cover (%) monthly values were subtracted from 100 to calculate possible sunshine (%) values.

### Vegetation simulations

Vegetation was simulated with LPJ [12], modified to use the Global Soil Data Task [23] multi-layer soil data set. Each vegetation simulation was run using a spin-up period of 800 years that consisted of repeated sets of 1901–1930 monthly temperature, precipitation and sunshine data. These spin-up data were detrended by applying locally weighted regressions [42] to estimate and subtract long-term trend values from the data (see Prentice *et al*. [43]). The spin-up period was followed by simulations using the historical (1901–2000) and projected future (2001–2099) climate data. Global mean annual atmospheric CO_2_ concentrations for the 21^st^ century were obtained from the Integrated Science Assessment Model (ISAM) reference case simulations for the A2 emissions scenario with 2070–2099 (30-year mean) atmospheric CO_2_ concentrations of 734 ppm [44]. LPJ was run using open multi-processing (OpenMP) commands (http://openmp.org) to parallelize the code for workstations with dual 6-core processors. Run times for individual simulations were on the order of hours to days depending on the simulation being run and equipment being used. Input and output files combined were approximately 1 TB in size for each simulation and the number of data reads and writes required for each simulation contributed to run time lengths.

**Fig 2:**
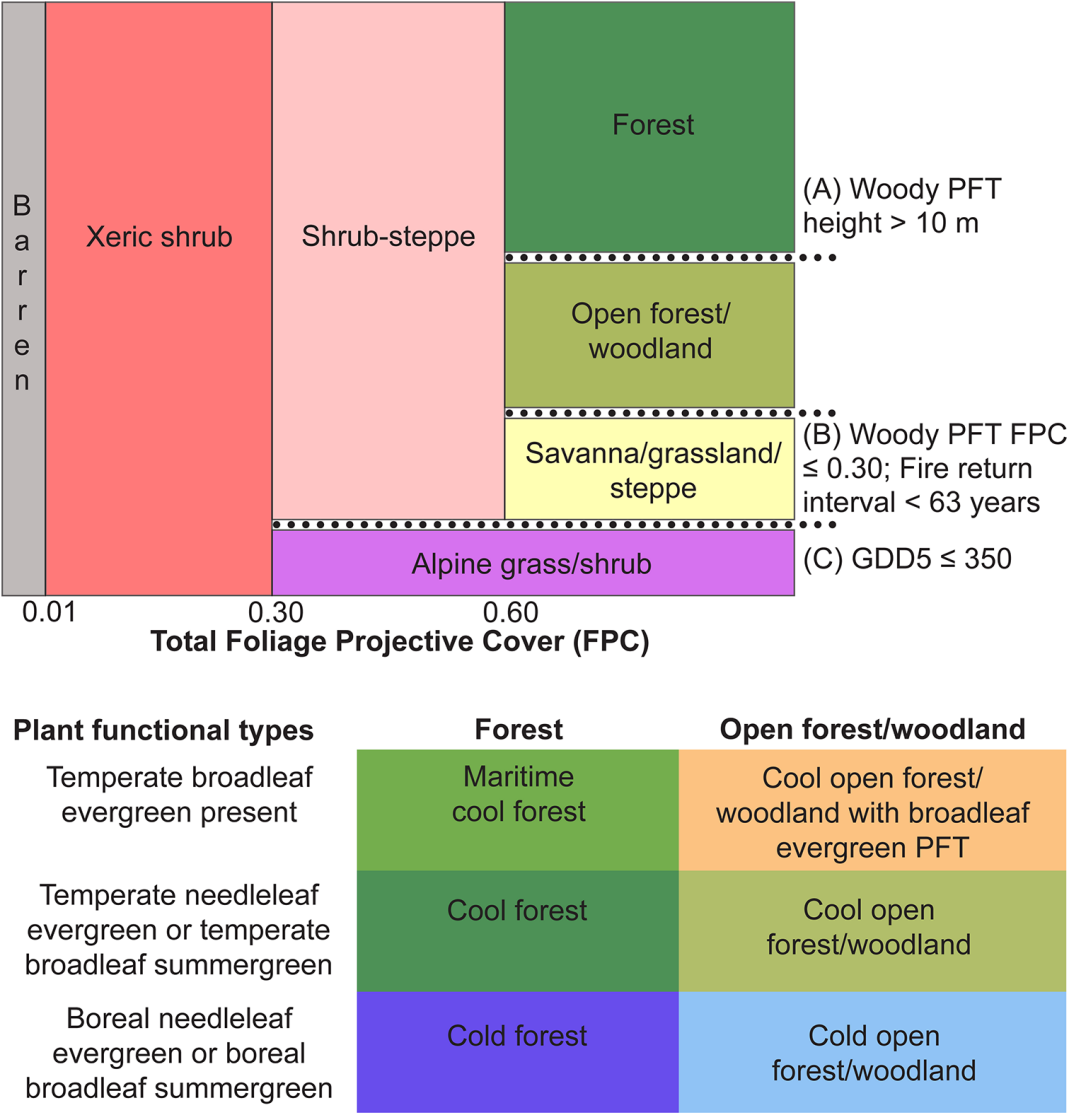
Biome assignment scheme for LPJ simulated vegetation. (A) Simulated woody plant functional type (PFT) height (m) is used to distinguish forest (>10 m) from open forest/woodland (≤10 m). (B) Savanna/grassland/steppe is assigned when woody PFT foliar projective cover (FPC) is ≤0.30 and the fire return interval is <63 years. (C) Alpine grass/shrub is assigned when annual growing degree days (5°C base; GDD5) are ≤350 (Table 2).

LPJ simulated a set of 10 PFTs (Table 2). PFT names and parameter values followed those of Sitch *et al*. [12]. PFT bioclimatic limits also followed Sitch *et al*. [12] with the exceptions listed in Table 2. The simulated PFTs were classified into 11 biome types using a combination of simulated PFT mean foliage projective cover (FPC; proportion of grid cell) and PFT mean height (m) as displayed in Fig 2. This method was modified from Prentice *et al*. [19]. A 30-year mean GDD5 value ≤350 degree days was used to identify alpine vegetation [19]. The savanna/grassland/steppe biome was defined as having a total tree PFT FPC ≤0.30. Thonicke *et al*. [45] evaluated the fire model used in LPJ and found that simulated fire return intervals were longer than observed fire return intervals for the grassland regions of central North America. Based on results described in Thonicke *et al*. [45], we used an LPJ-simulated fire return interval of 63 years in the biome assignment process to distinguish between open forest/woodland and savanna/grassland/steppe biomes (Fig 2, [46]). The LPJ-simulated fire return interval did not include the effects of direct anthropogenic fire ignition or suppression. We also did not impose anthropogenic land cover changes (e.g., agriculture, urbanization, logging) in our vegetation simulations.

**Table 2:** LPJ plant functional type life form and bioclimatic limits.

Plant Functional Type	Tc_min_ (°C)	Tc_max_ (°C)	GDD5_min_ (deg. days)	(Tw-Tc)_min_ (°C)
Tropical broadleaf evergreen	15.5	—	—	—
Tropical broadleaf raingreen	15.5	—	—	—
Temperate needleleaf evergreen	-19.0 (-2.0)	— (22.0)	900	—
Temperate broadleaf evergreen	-1.0 (3.0)	18.8	1200	—
Temperate broadleaf summergreen	-17.0	15.5	1200	—
Boreal needleleaf evergreen	-32.5	0.0 (-2.0)	600	—
Boreal needleleaf summergreen	—	-2.0	350	43
Boreal broadleaf summergreen	—	-2.0	350	—
Temperate herbaceous (C3)	—	15.5	—	—
Tropical herbaceous (C4)	—	—	—	—

Plant functional type bioclimatic limits: Tc_min_ = mean temperature of the coldest month minimum limit; Tc_max_ = mean temperature of the coldest month maximum limit; (Tw-Tc)_min_ = mean temperature of the warmest month minus mean temperature of the coldest month minimum limit; GDD5_min_ = growing degree days on a 5°C base minimum limit. The bioclimatic limits match those of Sitch *et al*. [12] except for those modified limits, for which the original values from Sitch *et al*. [12] are given in parentheses. Limits were modified based on regional species distributions and climate information from Thompson *et al*. [47,48]. All PFTs have a woody life form with the exception of the two herbaceous PFTs. Herbaceous, as used here, refers to vegetation with non-woody stems and includes grass.

### Evaluation of simulated vegetation

**Fig 3:**
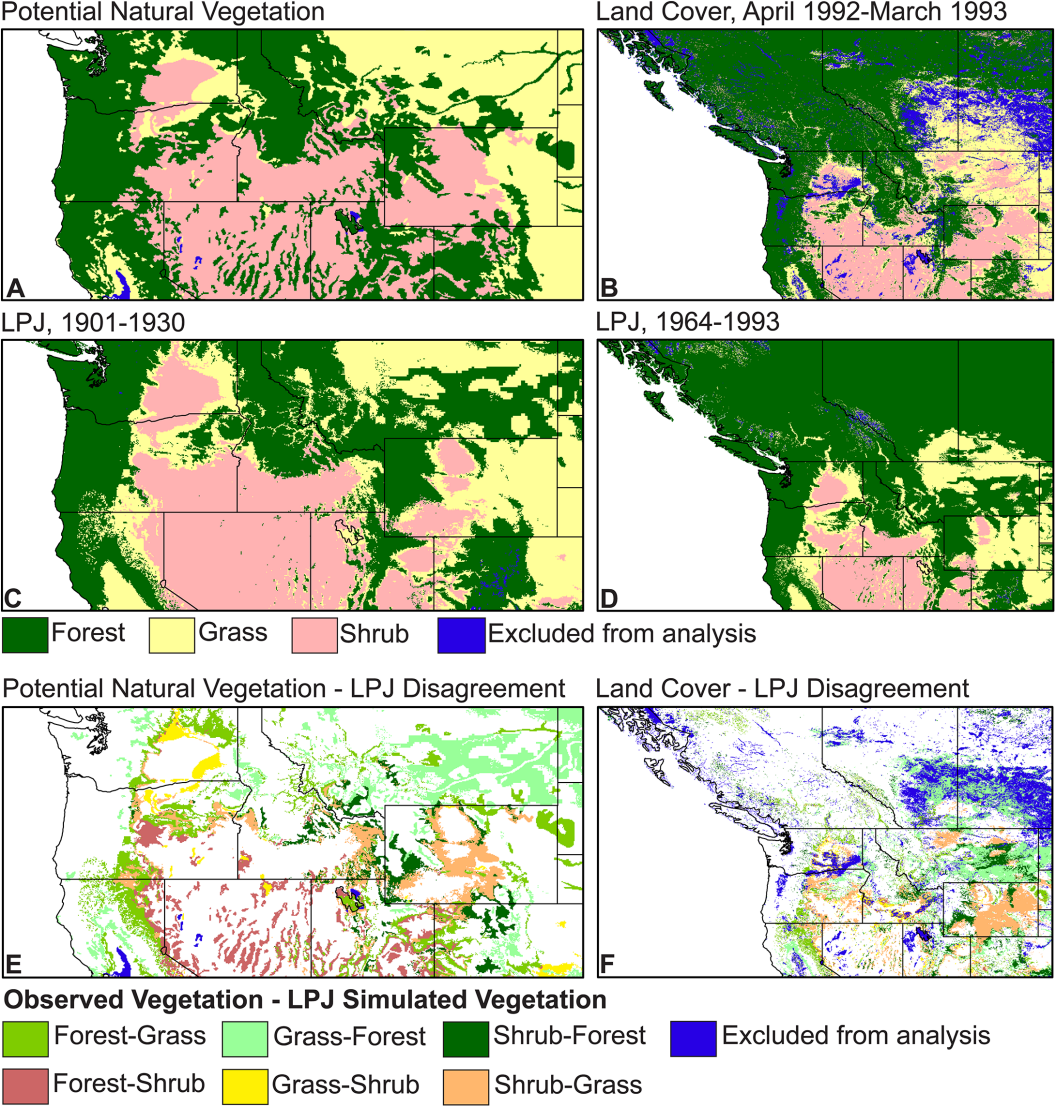
Evaluation of LPJ simulations. Evaluation was done using (A) Küchler [49,50] potential natural vegetation (PNV) data and (B) Advanced Very High Resolution Radiometer (AVHRR) land cover data from April 1992 to March 1993 [51]. Both data sets were reclassified into forest, grass, and shrub categories. Data excluded from analyses included PNV and land cover vegetation types not simulated by LPJ (e.g., agriculture, urban areas, wetlands). The PNV data were compared with (C) 1901–1930 [29] LPJ simulations and the land cover data were compared with (D) 1964–1993 [29] LPJ simulations, with (E, F) areas of disagreement displayed for each comparison. The legend indicates the type of disagreement. For example, the Forest-Grass category indicates areas where the (E) PNV or (F) land cover data recorded forest vegetation but LPJ simulated grass vegetation.

We compared the LPJ simulated 1901–1930 (30-year mean) vegetation with potential natural vegetation data for the United States [49,50]. The 28 Küchler vegetation forms and the LPJ simulated biomes were reclassified into forest, grass, and shrub categories (Fig 3; S1 Appendix). Since the Küchler [49,50] data set did not extend into Canada, we also evaluated the LPJ simulated vegetation with 1-km land cover data for North America derived from April 1992 to March 1993 Advanced Very High Resolution Radiometer (AVHRR) remotely sensed data [51]. The AVHRR-derived land cover types were reclassified into forest, grass, and shrub categories and compared with the LPJ simulated 1964–1993 (30-year mean) biomes, which were also reclassified into forest, grass, and shrub categories (Fig 3; S1 Appendix). We compared the reclassified simulated vegetation data for each grid cell with the reclassified observed Küchler or AVHRR remotely sensed vegetation for the same grid cell to calculate percent agreement for forest, grass, and shrub vegetation across the study area (Tables 3 and 4). Potential natural vegetation and land cover types not simulated by LPJ (e.g., agriculture, urban areas, wetlands) were excluded from the evaluation (S1 Appendix).

**Table 3:** Percent agreement of LPJ simulated vegetation with Küchler [49,50] potential natural vegetation (PNV) form.

	LPJ Biomes, 1901–1930 (30-year mean)		
Küchler PNV Form	Forest (%)	Grass (%)	Shrub (%)
Forest	69	19	11
Grass	30	63	5
Shrub	10	16	73

All data were classified into forest, grass, and shrub categories (see S1 Appendix).

**Table 4:** Percent agreement of LPJ simulated vegetation with Advanced Very High Resolution Radiometer (AVHRR) remotely sensed land cover data [51].

	LPJ Biomes, 1964–1993 (30-year mean)		
AVHRR Land Cover	Forest (%)	Grass (%)	Shrub (%)
Forest	93	5	1
Grass	48	46	5
Shrub	19	30	50

All data were classified into forest, grass, and shrub categories (see S1 Appendix).

## Results

### Simulated historical vegetation

The broad-scale spatial patterns of the simulated vegetation are visually quite similar to the observed vegetation data (Fig 3). Compared with Küchler’s [49,50] potential natural vegetation data for the United States, LPJ correctly simulates the vegetation at 69% of the grid points, including 69% of the forest grid points, 63% of the grass grid points, and 73% of the shrub grid points (Table 3). Compared with North America AVHRR remotely sensed land cover data [51] for the entire study area, LPJ correctly simulates the vegetation at 75% of the grid points, including 93% of the forest grid points, with lower agreement for the grass (46%) and shrub (50%) grid points (Table 4). For both the potential natural vegetation and land cover comparisons, observed grass and herbaceous biomes are the least well simulated by LPJ (Tables 3 and 4, S1 Appendix).

Significant areas of disagreement between observed and simulated vegetation occur in eastern Montana and southern Alberta and Saskatchewan, where LPJ simulates forest and woodland in areas classified as grass-dominated by both the Küchler [49,50] and land cover [51] data sets (Fig 3). There are also large areas where LPJ simulates savanna/grassland/steppe vegetation and the observed vegetation data sets record shrub vegetation. These areas primarily occur in central Wyoming, southeastern Idaho, and in eastern Oregon (Fig 3). The opposite pattern occurs along the northern and eastern edge of the Columbia Plateau, where the Küchler [49] potential natural vegetation data record grassland and LPJ simulates shrub-steppe. LPJ underestimates forest along the east slope of the Cascade Range and Sierra Nevada mountain ranges, the northeast edge of the Columbia Plateau, and for mountain ranges in the Great Basin of Nevada and Utah. Inspection of the locations and nature of the disagreements suggests that they are in part the result of the relatively coarse spatial resolution of the soil data, and the necessarily low taxonomic resolution of the reclassified vegetation data (both simulated and observed; S1 Appendix). However, these disagreements do not seriously limit the interpretability of the results.

### Simulated future vegetation

**Fig 4:**
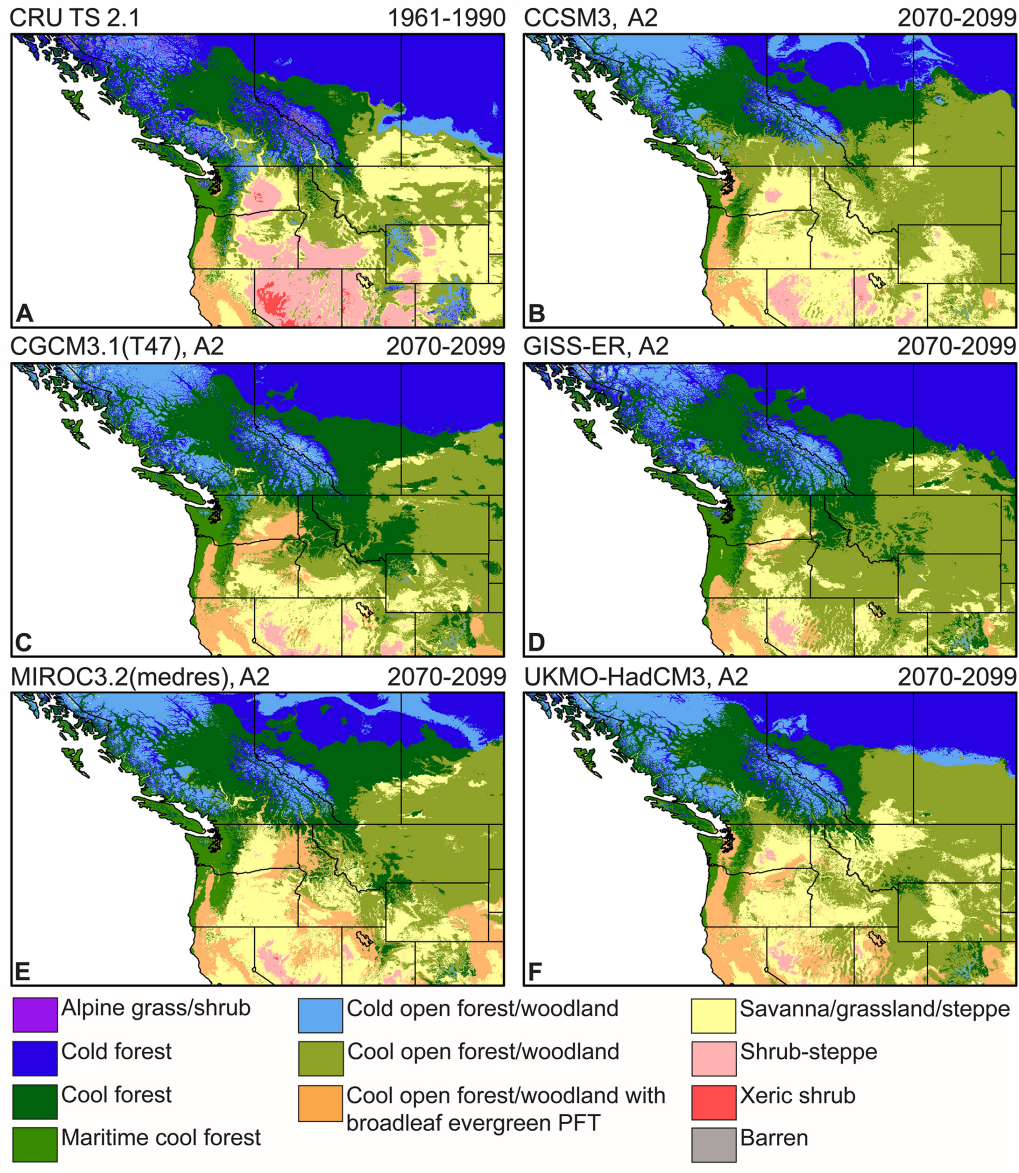
LPJ simulated vegetation. Vegetation was simulated for (A) 1961–1990 using CRU TS 2.1 climate data [29] and for (B-F) 2070–2099 using climate projections from CCSM3 [32], CGCM3.1(T47) [33], GISS-ER [34], MIROC3.2(medres) [35], and UKMO-HadCM3 [36] coupled atmosphere-ocean general circulation models (AOGCMs). PFT = plant functional type.

Under all five future climate projections, LPJ simulates substantial changes in vegetation across the study area (Fig 4, Table 5). At high elevations, cold forest, cold open forest/woodland, and alpine grass/shrub areas decrease or disappear and are replaced by cool forest and cool open forest/woodland, particularly at lower latitudes. This trend is particularly apparent in the Cascade Range and the Rocky Mountains in the United States. The southern boundary of cold forest in Canada is simulated to shift northward and is replaced by cool forest and cool open forest/woodland. Under all five future climate simulations, cold open forest/woodland is simulated to expand from 6.2% of the study area to 6.3–10.5%, particularly in British Columbia (Table 5). Under historical climate conditions, the interior parts of the study area were dominated by savanna/grassland/steppe, shrub-steppe, and xeric shrub vegetation [49]. Under simulated future climate conditions, large areas of shrub-steppe and xeric shrub vegetation are replaced by savanna/grassland/steppe vegetation. This replacement occurs throughout the interior Great Basin and Columbia Plateau with areas of shrub-steppe vegetation simulated to decrease in the study area from 10.5% to 0.6–2.4% by 2070–2099 (Table 5).

**Table 5:** Simulated biomes (% of study area grid cells) for 1961–1990 and 2070–2099 (bold text indicates biomes that are simulated to increase under projected future climates).

	1961–1990 (%)	2070–2099 (%)				
Biome	CRU TS 2.1	CCSM3	CGCM3.1(T47)	GISS-ER	MIROC3.2(medres)	UKMO-HadCM3
Alpine grass/shrub	0.6	<0.1	<0.1	<0.1	<0.1	<0.1
Cold forest	25.7	13.4	18.2	21.5	12.3	16.5
Cool forest	14.6	14.5	**25.0**	**22.6**	**21.2**	**15.1**
Maritime cool forest	3.1	**3.8**	**4.6**	**4.3**	**4.9**	2.7
Cold open forest/woodland	6.2	**9.3**	**8.2**	**6.3**	**10.5**	**9.9**
Cool open forest/woodland	17.3	**31.2**	**24.1**	**31.8**	**23.0**	**29.5**
Cool open forest/woodland with broadleaf evergreen plant functional type	2.5	**3.3**	**5.8**	**3.2**	**8.9**	**8.3**
Savanna/grassland/steppe	18.1	**21.9**	12.6	9.6	**18.2**	17.3
Shrub-steppe	10.5	2.4	1.3	0.6	1.1	0.6
Xeric shrub	0.7	<0.1	<0.1	<0.1	<0.1	0
Barren	0.6	<0.1	<0.1	<0.1	0	<0.1

In addition to the vegetation changes described above, there are also regions of the study area where the historical vegetation is simulated to persist under future climate conditions for 2070–2099 (Fig 4). Cold forest in parts of northern Alberta and Saskatchewan is simulated to remain unchanged. Large areas of simulated maritime cool forest along the coast of British Columbia, Washington, and Oregon, as well as at lower elevations in the Cascade Range, remain in the future simulations. Cool open forest/woodland with a broadleaf evergreen PFT component is also simulated to remain in parts of western Oregon and northern California. Overall, forest and woodland vegetation is simulated to increase from 69.4% in the 1961–1990 simulation to 75.5–89.7% under the five future 2070–2099 simulations (Table 5). Additionally, large areas of savanna/grassland/steppe vegetation in the northern part of the Sacramento Valley in California are simulated to persist under all five future climate simulations.

## Discussion

This study describes the application of LPJ at a 30-second spatial resolution for a large region of western North America. The results represent an advance over previous LPJ simulations for this region because they resolve fine-scale patterns of vegetation change that are not visible when the model is run at coarser spatial resolutions. Resolving these fine-scale vegetation patterns is particularly important for understanding the dynamics of future vegetation changes in regions of complex topography, such as our study area. The results of our evaluation support the use of LPJ at similar spatial resolutions for other regions of the globe.

### LPJ simulations of historical vegetation

**Fig 5:**
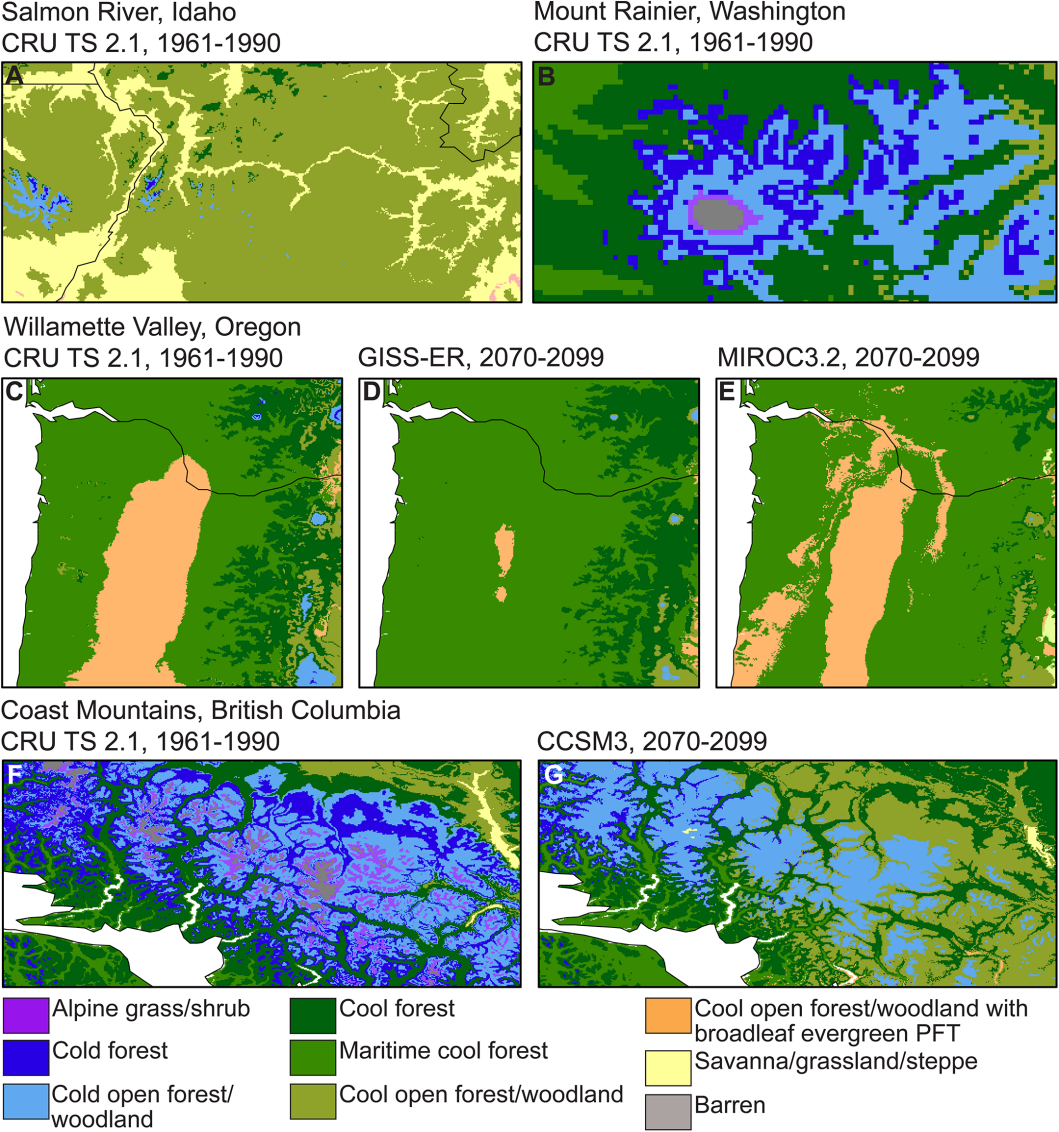
Regional examples of LPJ simulated vegetation. LPJ 1961–1990 [29] simulated vegetation for (A) the Salmon River region of Idaho and (B) Mount Rainier, Washington. (C) Willamette Valley, Oregon, historical (1961–1990 [29]) and projected future (2070–2099) vegetation as simulated by the (D) GISS-ER [34] and (E) MIROC3.2(medres) [35] coupled atmosphere-ocean general circulation models (AOGCMs). (F) Historical (1961–1990 [29]) and (G) simulated future (2070–2099, CCSM3 [32]) vegetation for the Canadian Coast Range. PFT = plant functional type.

LPJ does a good job of simulating the broad-scale patterns of forest, grass, and shrub vegetation across the study area (Fig 3; Tables 3 and 4). The simulated historical vegetation patterns are generally similar to those produced by other vegetation models for parts of the region at similar spatial resolutions (e.g., Rogers *et al*. [52]). The 30-second grid is able to resolve a number of fine scale vegetation patterns that are not visible at coarser spatial resolutions. Valley bottom vegetation is distinguishable in a number of areas, such as along the Salmon River in Idaho (Fig 5A). Also visible are vegetation transitions over elevation gradients in mountainous regions, such as along the slopes of Mount Rainier, Washington, where vegetation is simulated as transitioning along elevational gradients from cool forest, cold forest, and cold open forest/woodland to alpine vegetation above tree line at high elevations (Fig 5B). These transitions along elevation gradients generally match historical vegetation transitions in the region [53].

In some areas, the LPJ-simulated vegetation improves on previous DGVM simulations. For example, studies using DGVMs [52,54] have had difficulty resolving the mix of forest, woodland, and wetland prairie vegetation that historically was present throughout the Willamette Valley in Oregon [55]. LPJ, as implemented here, simulates open forest/woodland with a broadleaf evergreen PFT for the Willamette Valley (Figs 4A and 5C), which represents a relatively good match with the valley’s potential natural vegetation [49]. Although not the dominant vegetation, a number of broadleaved evergreen species are noted in the 1850s United States General Land Office surveys as being present in the Willamette Valley, including Pacific madrone (*Arbutus menziesii*) and manzanita (*Arctostaphylos* spp.) [55]. LPJ simulates this same open forest woodland with broadleaf evergreen PFT vegetation in the area around Puget Sound, Washington, and in the southeast part of Vancouver Island, British Columbia, where prairies and open woodlands, particularly oak woodlands, historically occurred [53]. This vegetation type also is simulated for parts of southern Oregon and northern California where it represents the more open forests and woodlands of this region, such as the broadleaf oak woodlands of northern California. Although fire ignition by Native Americans is often invoked as necessary for having maintained open vegetation in these regions prior to Euro-American settlement, anthropogenic ignitions are not simulated by LPJ. The simulated vegetation results imply that climate is sufficient for maintaining open vegetation, which is consistent with evidence from paleoenvironmental records that, regardless of the frequency of anthropogenic ignitions, climate played an important role in the maintenance of open vegetation in these regions [56].

Regions where LPJ-simulated vegetation does not match the observed vegetation occur primarily in the interior, more arid parts of the study area (Fig 3). In some cases, the simulated disagreement reflects the control exerted on vegetation by the soil data via soil moisture. For example, simulated cool open forest/woodland along the Missouri River in eastern Montana is overrepresented when compared with the Küchler [49] potential natural vegetation. In this case, the simulated vegetation follows the boundaries of the underlying soil type that occurs along the Missouri River in eastern Montana but over a larger area than the floodplain forest identified by Küchler [49], as can be seen by comparing Fig 3A and 3C. In other cases, the simulated disagreement may reflect limits in the ability of this version of LPJ to simulate fire. Fire is an important control on the distribution and dynamics of woodland, grass, and shrub vegetation across the study area. On the Columbia Plateau, low severity, high frequency fires historically occurred at lower elevations [57]. These types of fires are not well simulated by this version of LPJ [45] although recent work by Pfeiffer *et al*. [58] and Thonicke *et al*. [59] to improve LPJ simulation of fire may help to resolve these issues. Additionally, some of the disagreement between the simulated vegetation and the 1992–1993 land cover data [51] may reflect human activities that have affected vegetation. For example, areas where woodland and forest expansion has occurred on the landscape as a result of recent fire suppression and other human land-use activities would be recorded in the 1992–1993 remotely sensed land cover data but would not be simulated by LPJ [57].

Disagreement between simulated and observed vegetation also reflects the limitations of the 30-second grid’s ability to represent vegetation variations that occur at scales below the resolution of an individual grid cell. For example, at high elevations there are many alpine and subalpine meadows that may be smaller than an individual 30-second grid cell and will not be resolved by our study area grid. Topography also can vary significantly over relatively short distances, with changes in aspect and slope affecting climate and hence vegetation patterns. Additionally, soils may exert strong controls on vegetation distributions but some fine-scale vegetation patterns may not be simulated by LPJ because particular soil types are not resolved by the 30-second grid.

### LPJ simulations of future vegetation changes

The simulated expansion of vegetation in 2070–2099 occurs not only poleward in latitude and upward in elevation, but in all directions, including southward and to lower elevations, illustrating the diversity of potential vegetation responses to climate changes in regions of topographic complexity, such as our study area (Fig 5). Under all five future climate projections, LPJ simulates the expansion of forest and woodland vegetation across large parts of the central and eastern regions of the study area. In general, this response is similar to a continuation of historical woodland and forest dynamics in the interior western United States. Examples include the expansion of western juniper (*Juniperus occidentalis*) woodland in eastern Oregon, where climate changes as well as changes in disturbance regimes and land-use activities have played a role in woodland expansion [60]. Some of these factors, such as fire suppression, reduction in browser populations, and land-use changes, were not included in our vegetation simulations. Other factors, such as the physiological effects of changes in atmospheric CO_2_ concentrations, are simulated by LPJ and may alter plant water-use efficiency and plant available water, affecting the competitive interactions between PFTs and contributing to the simulated future invasion of grassland and shrubland vegetation by trees [11,21,61]. Another example of the complex response of vegetation to climate change is the simulated future expansion of maritime cool forest into the Willamette Valley, which is most pronounced under the GISS-ER and MIROC3.2(medres) simulations (Fig 5D and 5E). This expansion also matches recent historical patterns of forest expansion in this region [55]. However, LPJ also simulates a different response, the potential future expansion of open forest and woodland vegetation in the Willamette Valley under the CCSM3 and UKMO-HadCM3 simulated climates (Fig 4). These different simulated vegetation responses may reflect important differences in the AOGCM-simulated climates and illustrate the need for additional research to evaluate the processes and thresholds involved in simulated vegetation changes for this region.

Under all five future climate simulations, high-elevation tree lines are simulated to move upwards resulting in the contraction of alpine grass/shrub vegetation, such as in the Canadian Coast Mountains (Fig 5F and 5G). Increasing elevation of tree lines is consistent with tree line responses observed in the paleoecological record during past periods of temperature change, although tree line response can be quite variable depending on location [62]. Other factors, ranging from fire suppression and grazing to soil water availability and nutrients, also may affect tree line response [62]. Site-specific factors, such as localized wet meadows or herbivore grazing, may limit tree establishment and help some open areas to persist as climate changes. Note that in the current LPJ simulations we have not excluded vegetation from areas that are currently covered by perennial ice and snow, such as parts of the Canadian Rocky Mountains. Areas classified as barren at high elevations are regions of low productivity resulting from the combination of climate and soil conditions at those locations. Future vegetation changes in these areas may be limited by the rate of soil development.

Another vegetation change simulated for 2070–2099 under all five future climate simulations is the reduction in the area of shrub-steppe and xeric shrub in the interior of the study area and its replacement by savanna/grassland/steppe or open forest/woodland vegetation. This simulated reduction in shrub-dominated vegetation may reflect, in part, the effects of increased atmospheric CO_2_ concentrations on vegetation, which may increase plant water-use efficiency, allowing trees to expand into more arid parts of the study area occupied by shrubs [11,61]. This simulated vegetation change would have significant implications for many species in this region, such as Greater Sage-grouse (*Centrocercus urophasianus*), a species of conservation concern which relies on sagebrush (*Artemisia* spp.) shrub-steppe habitat.

### Comparison with other studies

Our simulated future vegetation changes are similar to those produced by other studies for the region, although direct comparisons are limited by differences in experimental protocol. Rogers *et al*. [52], using the MAPSS-CENTURY 1 (MC1) DGVM [63], simulated similar expansion of forest vegetation and reduction of shrubland for 2070–2099 in arid parts of eastern Oregon and Washington. They also used CMIP3 A2 future climate simulations at a 30-second spatial resolution, although other aspects of their experimental protocol differed from our study (e.g., different soil and historical climate data, a 1971–2000 historical climate base period) [52]. Their simulations produced forest in western Oregon and Washington that persisted under projected future climate conditions in general agreement with our simulations, although our classification of forest types differed (Fig 4, Table 5). Hamann and Wang [64] used a statistical model to simulate future ecological zones and tree species distributions for British Columbia, using an IPCC IS92a future climate simulation [65]. They simulated a 97% decrease in alpine tundra for their study area for 2071–2100, which agrees with our simulated decrease in alpine grass/shrub (Fig 5F and 5G). They also simulated an expansion of more arid ecosystems (e.g., bunch grass, ponderosa pine) into central British Columbia, which differs from our simulated expansion of cool forest in this region. This difference could be the result of a number of factors, including the physiological response of vegetation to changes in atmospheric CO_2_ concentrations, which is simulated by LPJ but not included in the statistical model results. Hamann and Wang [64] tested this possibility by increasing precipitation to approximate a CO_2_-driven change in water-use efficiency, which reduced the amount of arid ecosystem expansion and improved the agreement with our LPJ simulations.

### Uncertainties associated with interpreting vegetation simulations

There are a number of caveats that must be considered when interpreting these vegetation simulations. The simulated biome distributions provide important information about the potential dynamics and patterns of vegetation responses to climate change. These simulations should not, however, be interpreted as predictions of specific future changes that will occur at particular locations. There is still significant uncertainty regarding the rate, magnitude, and spatial expression of future climate change. As is clear from the biome simulations, different projections of future climate can result in significant differences in simulated vegetation patterns (Fig 4). We used five AOGCM simulations in this study but there are others that could be used. Giorgi [66] describes some of the uncertainties associated with model simulations of climate.

We have described relatively fine spatial resolution vegetation simulations that include significant topographic detail and help to characterize the potential spatial response of vegetation to future changes in climate and atmospheric CO_2_ concentrations. These simulations include a number of processes that are important in determining how vegetation responds to climate change [12]. However, there are important processes that affect vegetation type and distribution that are not simulated by this version of LPJ or that are simulated in a simplified form. Decisions of what processes to include in numerical vegetation models are driven by a number of factors, including what research questions are being investigated, the numerical efficiency (and speed) with which a model runs, and the availability of input data necessary to simulate processes at different scales. Understanding these limitations is important for accurately interpreting the simulated vegetation. We have already mentioned some of the processes not included in our simulations, such as human activities resulting in land cover changes. Other important processes include the effects of insect and disease outbreaks that may affect vegetation distribution patterns over time. For example, the simulated persistence of forest in Canada (Fig 4) does not include the potential future effects of bark beetle outbreaks that have had significant impacts on forests in western North America [67]. LPJ also does not explicitly simulate plant dispersal but instead assumes that dispersal rates will be fast enough to accommodate vegetation responses to climate change. Whether this assumption is appropriate will depend on a number of factors, such as potential future land-use activities that may create dispersal barriers and the extent to which human-assisted species migration may occur. It is important to note that the various model limitations discussed here are not unique to LPJ [9], and the model continues to be developed, including efforts to improve simulation of plant hydraulic architecture [68], peatland and permafrost dynamics [69], and hydrology [70].

Although we simulated vegetation for grid points spaced 30-seconds (~1-km) apart, there is a significant amount of environmental variability in the study area that occurs at finer spatial resolutions. Topographic and climatic controls on vegetation, such as fine-scale variations in slope and aspect, may have significant effects on the type and distribution of vegetation at local scales, and these fine-scale variations were not resolved by our 30-second study area grid. Local hydrology and the presence of various water bodies (lakes, reservoirs, etc.) may produce distinct wetland, riparian, and aquatic vegetation at sub-grid cell resolutions, such as wet meadows. However, the version of LPJ we used does not simulate the effect of wetland hydrology on vegetation (e.g., inundation stress) and we excluded wetland and aquatic habitat from our study (S1 Appendix).

There are also limits to the temporal resolution of our analysis. Important climate variability occurs on sub-monthly temporal scales and this temporal variability can affect vegetation patterns. Of particular importance to vegetation will be the future frequency and duration of extreme climate events (e.g., heat waves, droughts). There are still limits in the ability of climate models to accurately simulate these extreme events [71] and they are not well represented in the monthly climate data we used as input for our vegetation simulations. However, LPJ does estimate the effects of extreme events on vegetation, such as cumulative heat stress or mortality from extreme low temperatures [12].

In addition to spatial and temporal resolution limitations, our model simulations have a limited taxonomic resolution. We simulated potential future vegetation change in terms of biomes as these simulations provide important information about potential future distributions of major habitat types. PFT parameters in LPJ may be chosen to represent individual species, although this is more commonly done with models that resolve stand-level population dynamics and resource competition (e.g., LPJ-GUESS [72]) and are better able to simulate species interactions, such as competition for light and water at different successional stages [73]. Uncertainties associated with a number of LPJ's parameters are described by Zaehle *et al*. [74].

### Implications for conservation and natural resource management

The potential vegetation changes simulated for 2070–2099 are quite large (Fig 4). The magnitude of these changes may present challenges for land managers attempting to develop adaptive management responses to climate change. Our results indicate that DGVMs, such as LPJ, can relatively accurately simulate historical vegetation patterns at a spatial resolution commensurate with many managed areas, such as national forests in the United States or whole systems areas used by The Nature Conservancy. This accuracy in simulating historical vegetation patterns lends confidence to the ability of the model to simulate future vegetation changes. By providing projections of future vegetation changes over a large region, fine spatial-scale simulations may also contribute to conservation and natural resource management being carried out at local scales. Biomes, as simulated here, provide information on potential future vegetation changes in terms of important habitat types (e.g., forest, grassland). These results complement model simulations of plant taxa responses to climate change (e.g., Hamann and Wang [64], Coops and Waring [75], Rehfeldt *et al*. [10]) and may be particularly relevant for conservation strategies focused on preserving ecosystem structure and function [76].

Land managers engaged in responding to climate change may want to translate simulated vegetation changes and their accompanying uncertainties into management actions. This effort may include combining simulated vegetation changes, such as the LPJ simulations presented here, with the expert knowledge of land managers concerning their local ecosystems and species of management concern, and many efforts are underway to guide managers in this process (e.g., Cross *et al*. [77]). Key results from our study that may contribute to these efforts include the simulated persistence of forest biomes, particularly in the northern parts of the study area, accompanied by the expansion of cool biomes into areas previously occupied by cold biome types (Fig 4). This result indicates that certain areas may be able to maintain forest habitat under future climate conditions although the species composition of these habitats may change. Alpine vegetation is simulated to decrease throughout the study area, in agreement with other future vegetation change studies for this region (e.g., [52,64]). One of the largest vegetation changes identified in our study is the simulated expansion of woodland and forest into arid grassland and shrubland under projected future climate conditions. However, this simulated expansion of trees may reflect, in part, an underestimation of fire occurrence in our simulations for these regions, and these results may be improved by additional research.

## Supporting Information

S1 Appendix Classification of vegetation data into forest, grass, and shrub categories. (PDF)
