# Supplementary material for: Projected Future Vegetation Changes for the Northwest United States and Southwest Canada at a Fine Spatial Resolution Using a Dynamic Global Vegetation Model
Source: PLoS One. 2015 Oct 21;10(10):e0138759. doi: 10.1371/journal.pone.0138759 (PMC4619408; doi:10.1371/journal.pone.0138759)
Supplement: S1 Appendix — (PDF) [file pone.0138759.s001.pdf]

S1 Appendix. Classification of vegetation data into forest, grass, and shrub categories.

Potential Natural Vegetation Data

The Küchler (1964, Conservation Biology Institute 2012) potential natural vegetation (PNV) data were reclassified into forest, grass, shrub, and excluded categories as listed in the following table. PNV types that were not simulated by LPJ or did not occur in our study area were excluded from the analyses.

PNV Form Value	Definition	Forest, Grass, Shrub, or Excluded Classification
1	Pacific Northwest (PNW) Conifer Forest	Forest
2	California Conifer Forest	Forest
3	Rocky Mountain Conifer Forest	Forest
4	Great Basin/Southwest Forest	Forest
5	California Mixed Evergreen	Forest
6	California Chaparral	Grass
7	Great Basin Shrub	Shrub
8	Desert Shrub	Shrub
9	California Grassland	Grass
10	Great Basin Grassland	Grass
11	Alpine Meadow	Grass
12	Desert Grassland	Grass
13	Great Basin Shrub/Steppe	Shrub
14	Desert Steppe	Grass
15	Desert Savanna	Grass
16	Wetlands	Excluded from analysis
17	Shortgrass Prairie	Grass
18	North Mixed Grass Prairie	Grass
19	Tall Grass Prairie	Grass
20	Coastal Prairie	Grass
21	Tall Grass Savanna	Excluded from analysis
22	Northern Conifer Forest	Excluded from analysis
23	Northern Hardwoods	Excluded from analysis
24	Floodplain Forests	Forest
25	Eastern Hardwood Forest	Excluded from analysis
26	Southern Mixed Forest	Excluded from analysis
27	Southeastern Subtropical Forest	Excluded from analysis
28	South Mixed Grass Prairie	Excluded from analysis

AVHRR Land Cover Data

The North America Land Cover Characteristics data (National Center for Earth Resources Observation and Science, 2002) were reclassified into forest, grass, shrub, and excluded categories as listed in the following table. Land cover types that were not simulated by LPJ or did not occur in our study area were excluded from the analyses.

Land Cover Value	Definition	Forest, Grass, Shrub, or Excluded Classification
1	Urban and Built-Up Land	Excluded from analysis
2	Dryland Cropland and Pasture	Excluded from analysis
3	Irrigated Cropland and Pasture	Excluded from analysis
4	Mixed Dryland/Irrigated Cropland and Pasture	Excluded from analysis
5	Cropland/Grassland Mosaic	Grass
6	Cropland/Woodland Mosaic	Forest
7	Grassland	Grass
8	Shrubland	Shrub
9	Mixed Shrubland/Grassland	Shrub
10	Savanna	Grass
11	Deciduous Broadleaf Forest	Forest
12	Deciduous Needleleaf Forest	Forest
13	Evergreen Broadleaf Forest	Forest
14	Evergreen Needleleaf Forest	Forest
15	Mixed Forest	Forest
16	Water Bodies	Excluded from analysis
17	Herbaceous Wetland	Excluded from analysis
18	Wooded Wetland	Excluded from analysis
19	Barren or Sparsely Vegetated	Barren
20	Herbaceous Tundra	Grass
21	Wooded Tundra	Forest
22	Mixed Tundra	Forest
23	Bare Ground Tundra	Barren
24	Snow or Ice	Barren
255	Unlabelled Land Area	Excluded from analysis

LPJ Simulated Biomes

The LPJ simulated biomes were reclassified into forest, grass and shrub categories as listed in the following table.

LPJ Biome	Forest, Grass, or Shrub Classification
Alpine grass/shrub	Grass
Cold forest	Forest
Cold open forest/woodland	Forest
Cool forest	Forest
Maritime cool forest	Forest
Cool open forest/woodland	Forest
Cool open forest/woodland with broadleaf evergreen plant functional type	Forest
Savanna/grassland/steppe	Grass
Shrub-steppe	Shrub
Xeric shrub	Shrub
Barren	Excluded from analysis

References

Conservation Biology Institute. U.S. potential natural vegetation, original Kuchler types, v2.0 (spatially adjusted to correct geometric distortions); 2012. Available: http://databasin.org/datasets/1c7a301c8e6843f2b4fe63fdb3a9fe39.

Küchler AW. Potential natural vegetation of the conterminous United States. American Geographical Society, Special Publication No. 36; 1964.

National Center for Earth Resources Observation and Science, U.S. Geological Survey. North American Land Cover Characteristics – 1-Kilometer Resolution; 2002. Database: National Atlas of the United States [Internet]. Available: http://nationalatlas.gov/.
